# An exploration into the application of specialty-orientated CBL pedagogy in undergraduate teaching in pediatric surgery

**DOI:** 10.3389/fped.2022.948853

**Published:** 2022-11-04

**Authors:** Wenyue Ma, Hongjie Gao, Xiaoya Liu, Mengmeng Chang, Changlin Jia, Fengyin Sun

**Affiliations:** ^1^ Department of Pediatric Surgery, Qilu Hospital of Shandong University, Jinan, China; ^2^ Department of Pediatrics, Qilu Hospital, Cheeloo College of Medicine, Shandong University, Jinan, China

**Keywords:** CBL pedagogy, undergraduate teaching, pediatric surgery, application, exploration

## Abstract

**Objective:**

This study aims to identify whether the specialty-oriented case-based learning (CBL) pedagogy contributes to the teaching of basic theory and practical operation in undergraduate clinical teaching in pediatric surgery, and to assess the satisfaction of undergraduates.

**Methods:**

A total of 72 undergraduates in Grade 2016 who interned at Qilu Hospital of Shandong University were enrolled in this study. All these undergraduates voluntarily participated in this experimental study. They were randomly divided into the experimental group (the CBL group, *n* = 36) and the control group [the traditional lecture-based learning (LBL) group, *n* = 36] with the assistance of random number tables. In the control group, a traditional pedagogy was adopted and the knowledge in the textbook was explained according to the syllabus. In the experimental group, a specialty-oriented CBL pedagogy was adopted under the guidance of clinical instructors. After the teaching, a comparison was drawn between both groups in respect of the theoretical exam and practical exam scores. In addition, the teaching results were evaluated by a questionnaire survey.

**Results:**

The average theoretical exam scores and comprehensive scores of undergraduates in the CBL group were higher than those in the LBL group (*P* < 0.05). There was no significant difference in the practical exam scores between the CBL group and the LBL group (*P* > 0.05). However, those undergraduates in the CBL group attained higher scores in doctor-patient communication and perioperative diagnosis and treatment (*P* < 0.05). According to the questionnaire survey, the undergraduates in the CBL group had higher satisfaction than those in the LBL group. Besides, this specialty-oriented CBL pedagogy had higher performance in improving their ability to solve problems independently and cultivating and expanding their knowledge compared with the traditional pedagogy. Meanwhile, this specialty-oriented CBL pedagogy can cultivate the critical thinking of undergraduates, which could increase their learning efficiency and improve their interest in learning.

**Conclusion:**

This specialty-oriented CBL pedagogy could improve the mastery of professional knowledge, course satisfaction, doctor-patient communication ability in clinical practice, and perioperative diagnosis and treatment ability of these undergraduates. Therefore, it is worthwhile to recommend and popularize this pedagogy in undergraduate clinical teaching in pediatric surgery.

## Introduction

Pediatric surgery is a frontier discipline that intersects with other basic medicine and clinical medicine disciplines. It includes pediatric general surgery, pediatric urology, pediatric orthopedic surgery, pediatric emergency surgery, neonatal surgery and other sub-professional disciplines. Currently, pediatric surgery teaching in China is mainly provided for undergraduates in pediatrics and clinical medicine in medical colleges. This program is routinely conducted in the form of theoretical knowledge in their fourth academic year and in the form of practice teaching of pediatric surgery in their fifth academic year during the internship. In the current pediatric surgery teaching, the traditional classroom teaching still predominates in both theoretical teaching and clinical practice and teaching. This traditional lecture-based pedagogy is performed as per the syllabus. As is revealed in some studies, there is insufficient instruction in traditional clinical teaching courses, which induces deficiencies in training the critical reasoning thinking of undergraduates in clinical teaching. However, it shall be noted that a critical thinking ability is necessary for clinicians (1).

CBL, also called inquiry-based learning, is a pedagogy originating from Harvard Law School based on problem-based learning (PBL) (2). Different from traditional lecture-based pedagogies that are used to conduct teaching based on repetition and memory (3), CBL is commonly employed to refine the knowledge into the cases in the form of human simulation by a rewriting method based on real cases in clinical practice (4–7). Subsequently, undergraduates are instructed to conduct group discussions, and hence they can devote themselves to clinical cases and the diagnosis and treatment environment can be favorably simulated. On that basis, the interest of undergraduates can be improved, and their clinical diagnosis and treatment ability and critical thinking can be cultivated.

Pediatric surgery teaching is mainly performed from basic theoretical knowledge and clinical practice. It is mainly characterized by abstract and complex teaching contents, complicated knowledge points, strong logic, and fewer teaching hours. Meanwhile, it also involves various diseases that are less clinically encountered by undergraduates. Therefore, it is difficult for undergraduates to master this discipline, and their learning initiative is not high. They are prone to learn this discipline by rote, which results in a poor learning effect. It shall be noted that pediatric surgery is not a reduced version of adult surgery. It includes distinct disease spectra and systematic treatment methods. Although the majority of pediatric surgical diseases can be divided into the same disease category as adult diseases, there are significant differences in the diagnosis and treatment schemes between both. According to some scholars' opinions, due to the distinct characteristics of the clinical knowledge system in pediatric surgery, the specialty-oriented CBL pedagogy shall be adopted in the undergraduate teaching of pediatric surgery and the differences between pediatric diseases and corresponding adult diseases shall be covered (8). Therefore, it is necessary to carry out specialty-oriented pediatric clinical surgery teaching and train professional pediatric surgeons.

Moreover, there is an uneven distribution for pediatric surgery resources in Shandong province, and the setting level of pediatric surgery varies significantly among different places. In many hospitals in prefecture-level cities, pediatric surgeons are served by clinicians in the adult surgery department. Additionally, those pediatric surgeons in many hospitals with pediatric surgery have no pediatric origins. These facts contribute to the nonstandard diagnosis and treatment of pediatric surgical diseases (9).

In recent years, most undergraduate medical students are employed by hospitals in prefecture-level cities. They are engaged in adult surgery and prone to serve as pediatric surgeons (10). Moreover, for the reason that only two academic weeks in pediatric surgery are arranged in the syllabus, these adult surgeons have little knowledge of pediatric surgery. Hence, it is a realistic and significant topic to master the knowledge of pediatric surgery that is closely related to adult surgery through short-term practice. In this study, the specialty-oriented CBL pedagogy would be introduced into the teaching practice reform of pediatric surgery. This pedagogy could achieve favorable teaching results based on a comparison with the traditional teaching mode. It would be reported in detail as follows.

## Data and methods

### General data

A total of 72 undergraduates in Grade 2016 from Shandong University who interned in the Pediatric Surgery Department of Qilu Hospital of Shandong University from June 2020 to July 2020 were selected as the research objects. All these undergraduates (including 44 males and 28 females) voluntarily participated in this experimental study. They were randomly divided into the CBL group and the LBL group with the assistance of random number tables, with 36 participants in each group. These undergraduates did not systematically acquire the theoretical knowledge related to pediatric surgery in their previous studies, and they also did not receive CBL teaching in their previous courses. In the CBL group, a specialty-oriented CBL pedagogy was adopted, including 24 males and 12 females aged (22.45 ± 0.91) years; In the LBL group, a traditional pedagogy was adopted, including 20 males and 16 females, aged (22.55 ± 0.85) years. The two groups of undergraduates are comparable in age and gender distribution (*P* > 0.05) (Table 1). At the end of the internship, all undergraduates were assessed in respect of the theoretical knowledge and practical operation of pediatric surgery. Besides, they also filled out questionnaires related to the satisfaction with this course. All participants signed the informed consent. This study was approved by the System Review Committee and Ethics Committee of Qilu Hospital of Shandong University.

**Table 1:** The basic characteristics of all the students.

Item	CBL group (*N* = 36)	Traditional group (*N* = 36)	Statistics	*P*
Age (y)	22.45 ± 0.91	22.55 ± 0.85	*χ*^2^ = 0.015	0.864
Gender				
Male	24	20	χ^2^ = 0.332	0.657
Female	12	16	χ^2^ = 0.135	0.463
Class cadres				
Yes	4	10	χ^2^ = 0.311	0.587
No	32	26	χ^2^ = 0.405	0.655

### Methods

They were randomly divided into the CBL group (*n* = 36) and the LBL group (*n* = 36). In addition, the clinical instructors were also divided into the experimental group (*n* = 5) and the control group (*n* = 5). In the LBL group, a traditional pedagogy was adopted and the knowledge in the textbook was explained according to the syllabus. In the CBL group, a specialty-oriented CBL pedagogy was adopted under the guidance of clinical instructors. The teaching steps were presented as follows.

A specialty-oriented CBL teaching plan is established to cover the differences between pediatric diseases and corresponding adult diseases.The diseases of pediatric surgery include hydrocele, indirect inguinal hernia, traumatic fracture, hydronephrosis, biliary tract dilatation, etc. The diagnosis, disease classification, treatment methods, treatment principles and surgical indications of such diseases are different from those of adults.The case selection for this study is based on typical cases with the same disease name in pediatric surgery and adult surgery, but the cases are quite different in etiology, diagnosis, and treatment. Taking an indirect inguinal hernia as an example, it has the following characteristics: (1) The cause of an indirect inguinal hernia in children is the developmental deformity of the sheath-like process, while the cause in adults is weakness or defect of the abdominal wall muscle. (2) The age of surgery for an indirect inguinal hernia in children is usually after 1 year (3) The surgical method for pediatric indirect inguinal hernia is high ligation of hernia sac, while for adults, tension-free repair of indirect inguinal hernia is required.. In the research, in order to guide students to access materials more efficiently and broaden their horizons, we provide students with 10 reference papers, in order to make up for the lack of students who have not studied medical retrieval courses due to their lower grades.

Combined with adult professional diseases, professional-oriented teaching and training of pediatric surgery can maximize the enthusiasm of students, exert their subjective initiative, and finally achieve the expected teaching effect. The qualified training of undergraduate students is the primary goal of undergraduate teaching reform.

The pedagogies in the experimental group were presented as follows. A teaching case was selected, and the course video and supplementary materials prepared by the instructor before class were distributed. These undergraduates received guidelines for the diagnosis and treatment of related diseases (Chinese and English versions), 10 reference papers related to course topics, and video materials of practical operation procedures of related diseases. Subsequently, these undergraduates previewed these study materials before class. In class, these undergraduates read cases to understand them and discuss them in groups after the introduction of clinical instructors. Then, these undergraduates put forward questions under the guidance of clinical instructors, which were assigned after the selection of clinical instructors according to the syllabus. After the first class, these undergraduates conducted the discussion in their groups with the aid of WeChat. In this process, they were encouraged to consult materials on the Internet or through books in the library, with a view to expanding their thinking. After obtaining the discussion results, they finally made a report PPT. In the second class, these undergraduates presented their reports through PPT. After that, they were provided with a mini clinical exercise evaluation (Mini-CEX). Through the “real-time feedback” in the evaluation, relevant problems were fed back to these undergraduates, so that they can realize the defects of their basic theoretical knowledge and skills in time. Meanwhile, the teaching level of clinical instructors was also enhanced.

The pedagogies in the control group were presented as follows. Before class, the instructors selected a case that was the same as that of the experimental group, and formulated a case plan. These undergraduates only conducted routine previews of knowledge. In class, the instructors explained the case and background knowledge in detail, and these undergraduates attended the class in groups without discussion. Instructors shall take a leading role in traditional lecture-based teaching.

**Figure 1:**
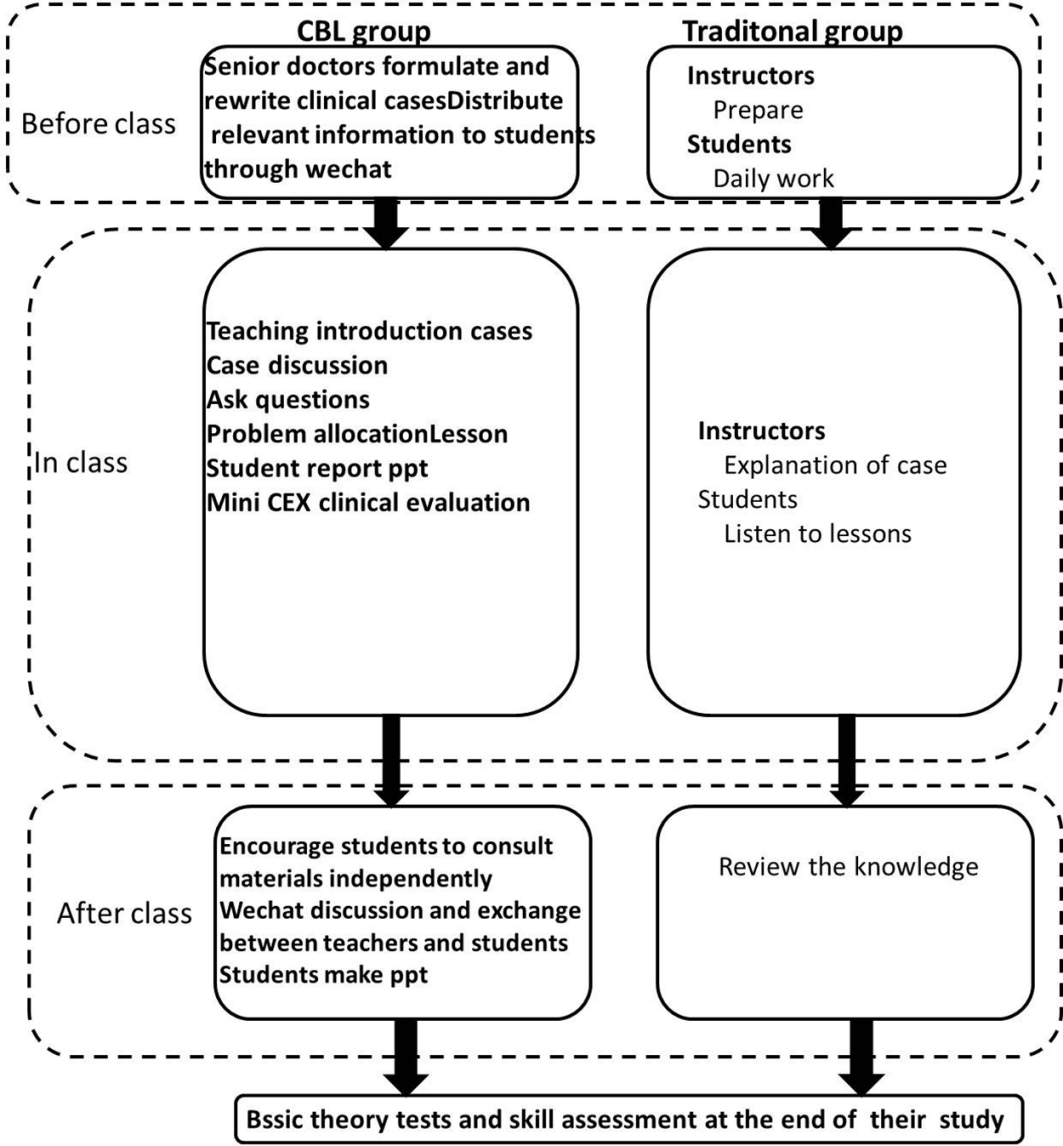
Teaching flow chart of CBL group and traditional teaching group.

All undergraduates signed the informed consent form, and they voluntarily participated in relevant exams and surveys. For the reason that the exam papers and questionnaires were labeled with their ID No. instead of their real names, the results of the exam papers and questionnaires had no (positive or negative) impact on their course scores or grades. These undergraduates completed relevant exams and surveys independently, without the assistance of their peers and instructors. The teaching process of the CBL group and the LBL group is shown in Figure 1.

After class, all undergraduates were asked to take a small exam related to theoretical knowledge and clinical practice, and all instructors and undergraduates were asked to fill out questionnaires.

### Data evaluation and statistical analysis

In this study, the clinical practice of undergraduates and the clinical teaching of instructors in the Pediatric Surgery Department of Qilu Hospital of Shandong University were evaluated through a basic theoretical exam, a clinical practice skill exam, and satisfaction questionnaire surveys. The assessment of clinical theoretical knowledge is carried out by means of examination papers, mainly using long case questions (LCQ), structured case questions (SCQ), modified case questions (MCQ), and short answer questions (SAQ). The clinical practice skills test is scored by the improved Mini-Cex evaluation system, and each score includes 1–10 points, including: consultation, medical record writing, physical examination, surgical operation, doctor-patient communication, perioperative diagnosis and treatment, and finally in After the practical skills are converted into a percentage system, the two test scores are added together to obtain a total score. The evaluation questionnaires related to satisfaction contained the overall satisfaction, teacher-student communication satisfaction, learning interest satisfaction, autonomous learning ability improvement satisfaction, learning efficiency satisfaction, knowledge mastery satisfaction, clinical logical thinking and clinical critical thinking cultivation satisfaction, learning burden reduction satisfaction, learning depth satisfaction, and learning breadth satisfaction. These undergraduates were evaluated from the above aspects. The same anonymous questionnaire was administered to both groups of students to assess their perceptions and experiences. The after-school survey includes 10 questions: learning motivation, self-learning ability, ability to solve problems independently, expanding knowledge, critical thinking, teamwork spirit, learning efficiency, reducing learning burden, and improving learning interest. According to the degree of improvement, the evaluation is divided into two grades: 1 = satisfied, 2 = dissatisfied.

Because this research relies on the daily teaching activities of Shandong University Clinical School of Medicine and the training plan for students, it is related to the students' academic performance in the future. Therefore, we cannot conduct a double-blind experiment on the evaluator's side, but can only conduct a single-blind experiment on the evaluator's side.

All statistical analysis was conducted by SPSS 24.0 software. The measurement data were expressed by mean ± standard deviation, and the significance was evaluated by the independent sample t-test. The enumeration data were subject to the Chi-square test to compare the teaching results between both groups. *P* < 0.05 indicated that the difference was statistically significant.

## Results

**Figure 2:**
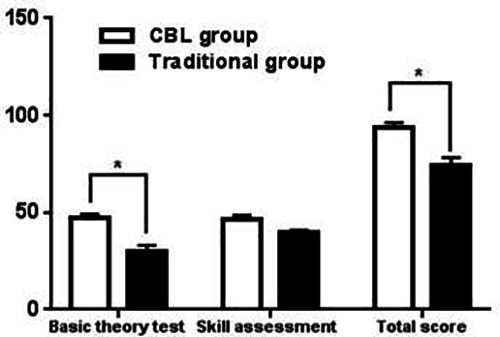
Average scores of basic theory test skill assessment and total score in the two groups.

The basic theoretical knowledge exam scores of the undergraduates in the CBL group were significantly higher than those in the LBL group (*P* < 0.05). Besides, the total scores of the CBL group were significantly higher than those in the LBL group (*P* < 0.05). It indicated that the CBL pedagogy had a positive effect on the mastery of basic theoretical knowledge and the improvement of total scores (Table 2, Figure 2).

**Table 2:** Graduation examination results.

Group	CBL group	Traditional group	T	*P*
Basic theory test	47.50 ± 2.05	41.45 ± 1.52	3.291	*P* < 0.05
Skill assessment	45.22 ± 2.80	44.60 ± 1.38	1.423	*P* > 0.05
Total score	90.32 ± 3.33	83.14 ± 2.0	3.765	*P* < 0.05

**Figure 3:**
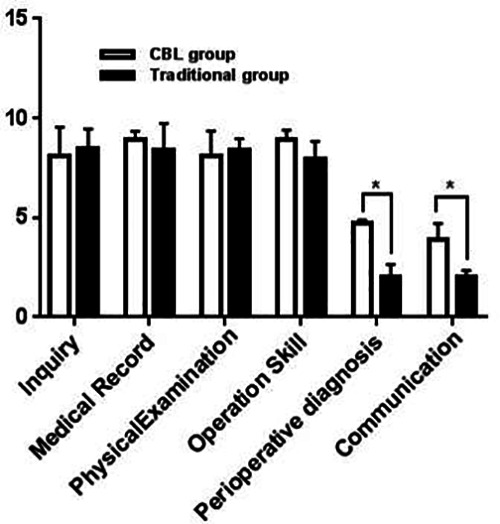
Average scores of various indicators of skill assessment.

Compared with the LBL group, the undergraduates in the CBL group scored higher in perioperative diagnosis and treatment and doctor-patient communication (*P* < 0.05). It suggested that the CBL pedagogy had a positive effect on cultivating the doctor-patient communication skills and perioperative diagnosis and treatment ability (Table 3, Figure 3).

**Table 3:** Skill assessment item results.

Skill assessment item	CBL group	Traditional group	T	*P*
Inquiry	7.30 ± 1.05	7.46 ± 0.96	1.070	0.171
Medical Record	8.40 ± 1.22	7.93 ± 1.37	1.321	0.132
PhysicalExamination	8.54 ± 1.46	8.67 ± 1.07	1.237	0.145
Operation Skill	7.23 ± 1.35	8.06 ± 0.74	1.434	0.108
Perioperative diagnosis	4.63 ± 1.72	2.63 ± 1.83	2.759	<0.05
Communication	4.33 ± 0.73	3.83 ± 1.64	2.043	<0.05

**Figure 4:**
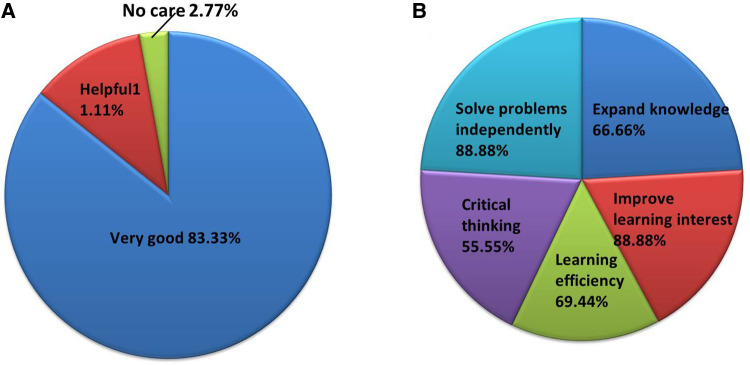
Students’ partial feedback on the questionnaire. (**A**) Attitude towards CBL teaching method; (**B**) Capacity improvement projects.

The overall satisfaction of the undergraduates in the CBL group was higher than that in the LBL group (*P* < 0.05). In addition, these undergraduates thought that the specialty-oriented CBL pedagogy could favorably enhance the independent problem-solving ability, expand knowledge, cultivate critical thinking, foster learning interest, and improve learning efficiency (*P* < 0.05) (Table 4, Figure 4).

**Table 4:** Students’ questionnaire survey results.

Item	CBL group (*N* = 36)	Traditional group (*N* = 36)	**χ** ^2^	*P*
Overall satisfaction	31	17	3.441	**0**.**032**
Sudy independently	32	18	0.681	0.062
Solve problems independently	32	16	2.533	**0**.**036**
Expand knowledge	24	10	2.567	**0**.**033**
Critical thinking	20	6	3.467	**0**.**022**
Teamwork spirit	17	13	0.532	0.073
Learning efficiency	25	15	1.954	**0**.**043**
Reduce thelearning burden	20	12	0.672	0.062
Improve learning interest	32	14	3.678	**0**.**013**

## Discussion

In this study, the basic theoretical knowledge exam scores of the CBL group and the LBL group (Table 2) clearly suggest that the CBL pedagogy can significantly improve these undergraduates’ mastery of theoretical knowledge. In the specialty-oriented CBL classes, the instructors would not blindly impart clinical knowledge to these undergraduates, and these undergraduates would not blindly acquire knowledge. A more realistic medical scene established by specialty-oriented CBL pedagogy encourages undergraduates in employing their subjective initiative to transform from the paradigm of “what I need to learn” to that of “what I want to learn”. As a result, these undergraduates can devote themselves to the learning (11), and they can truly experience clinical diagnosis and treatment in all directions. Meanwhile, since these undergraduates are encouraged to consult literature and materials for knowledge sharing, they can increase their learning depth and breadth, and relevant professional knowledge can be favorably mastered. It suggests that the specialty-oriented CBL pedagogy is a better teaching approach to the acquisition of basic theoretical knowledge.

At the same time, the clinical practice exam scores of the CBL group and the LBL group clearly indicate that there is no significant difference in the overall clinical practice scores between both groups (*P* > 0.05). It can be explained that there is a lack of experience in clinical practice in pediatric surgery for these undergraduates in both the CBL group and the LBL group. Thus, they cannot make complete and correct admission consultation and medical record writing for the disease in the case. Besides, they cannot make a systematic and correct physical examination for SP patients. There are distinct characteristics in pediatric surgery courses compared with the courses related to adult diseases. The teaching of clinical practice related to pediatric surgery becomes more difficult due to the complex anatomical structure of most organs in children, limited surgical space and prolonged learning cycle. Moreover, some scholars have conducted competency-oriented CBL courses in the clinical practice of pediatric surgery, and the results demonstrate that this pedagogy contributes to the teaching of clinical operation (12). It is required to continue to explore and improve this method. Therefore, the clinical practice is further analyzed in this study. The results indicate that the ability of doctor-patient communication and perioperative diagnosis and treatment of the undergraduates in the CBL group is significantly improved. It suggests that the unique and innovative pedagogy in the specialty-oriented CBL courses can fully train their clinical thinking. Thus, they can cope with various clinical changes in the perioperative diseases related to pediatric surgery. Further, this pedagogy can effectively cultivate their thinking related to disease diagnosis and treatment from the standpoint of a pediatric surgeon. Due to the fact that these undergraduates are required to actively communicate with patients in the specialty-oriented CBL course, their doctor-patient communication ability is significantly improved, which would benefit their clinical practice.

According to the satisfaction questionnaire survey results of both the CBL group and the LBL group, the undergraduates in the CBL group are more satisfied with their pedagogies. Under the real medical scenes in all directions in the classroom and the Mini-CES pedagogy, they can solve problems independently as surgeons. Hence, most undergraduates can improve their ability to solve clinical problems independently. On the ground that the instructors in the CBL group encourage students to consult reference materials independently in their spare time in the teaching process, these undergraduates' knowledge is broadened, and the breadth and width of learning are increased. Additionally, their interest in learning is improved by making a transformation from the passive acceptance to the active acquisition of theoretical knowledge. However, based on the fact that these undergraduates have received self-study-oriented open education and have certain self-study abilities, this specialty-oriented CBL pedagogy may have negligible effects on improving their self-study ability. In addition, the case discussion and analysis in class and the instructor-undergraduate and undergraduate-undergraduate discussion on WeChat after class could significantly improve their critical ability through this specialty-oriented CBL pedagogy. Meanwhile, their clinical diagnosis and treatment thinking is also improved, which is also reflected in the above-mentioned clinical practice scores, especially in the perioperative diagnosis and treatment scores. Moreover, some innovative pedagogies contribute to the significant improvement of their learning efficiency. For instance, 1. the clinical instructors can push pediatric surgery knowledge with maternal and child specialty characteristics on the WeChat platform every week, and present typical cases for discussions on the WeChat platform; 2. Mini-CEX can be conducted to significantly improve their learning efficiency. It shall be noted that the input time of these undergraduates increases, and the CBL pedagogy has no significant positive effect on reducing their learning burden due to the characteristics of the professional knowledge in pediatric surgery; However, a report on the CBL clinical pedagogy by Zhao et al. suggests that CBL can significantly reduce the learning burden on students (13). Therefore, it is necessary to continue to improve these learning methods and conduct more in-depth explorations.

As a key clinical department in Shandong province, the Pediatric Surgery Department of Qilu Hospital of Shandong University adopts the specialty-oriented CBL pedagogy in the teaching of undergraduates, including thoracic surgery, general surgery, urology, orthopedics and other sub-departments. Although this study is only continued 2 weeks, it has achieved favorable results in the exam assessment scores and the teaching satisfaction questionnaire survey. The reform of undergraduate courses in pediatric surgery can be conducted based on this specialty-oriented CBL pedagogy. Based on the cases in relevant departments, these undergraduates can understand the combination and intersection points of their major and pediatric surgery in clinical practice. On that basis, they can acquire comprehensive medical knowledge and improve their clinical diagnosis and treatment ability in clinical practice. However, only several options (satisfaction, normal and dissatisfaction) are designed for these instructors and undergraduates in the teaching satisfaction evaluation scale. Thus, only a rough estimate is obtained from this survey. The Likert scale may be more effective in evaluating the satisfaction of these instructors and undergraduates. Furthermore, there is no significant overall improvement in operation in clinical practice. Hence, this specialty-oriented CBL pedagogy shall be improved, in an attempt to impart more specific practical operation in pediatric surgery to the undergraduates.

This specialty-oriented CBL pedagogy could improve the mastery of professional knowledge, course satisfaction, doctor-patient communication ability in clinical practice, and perioperative diagnosis and treatment ability of these undergraduates. Therefore, it is worthwhile to recommend and popularize this pedagogy in undergraduate clinical teaching in pediatric surgery.

## Data Availability

The raw data supporting the conclusions of this article will be made available by the authors, without undue reservation.
